# Clinically- versus serologically-identified varicella: A hidden infection burden. A ten-year follow-up from a randomized study in varicella-endemic countries

**DOI:** 10.1080/21645515.2021.1932217

**Published:** 2021-06-28

**Authors:** Paul Gillard, Michael Povey, Stephane Carryn

**Affiliations:** GSK, Wavre, Belgium

**Keywords:** VZV, varicella, burden of infection, subclinical varicella, VZV seroprevalence

## Abstract

Varicella-zoster virus (VZV) infections cause a substantial disease burden, which is underestimated due to incomplete reporting data and lack of serological surveillance. In this post-hoc analysis of a randomized, Phase IIIb clinical trial (NCT00226499) with a ten-year follow-up period, we report anti-VZV antibody levels and persistence in non-vaccinated children, as a varicella infection estimate in ten European countries with endemic varicella. The present analysis specifically focuses on clinical and serological data from the control group, which included 827 healthy participants aged 12–22 months who received two doses of measles-mumps-rubella (MMR) vaccine. The per-protocol cohort included 744 children for whom varicella occurrence was evaluated by clinical definitions, epidemiological links and PCR test outcomes. Anti-VZV antibody levels were assessed by ELISA. The primary objective of this analysis was to correlate varicella occurrence with anti-VZV antibody levels. Varicella was confirmed in 47% of MMR recipients. Among participants without reported varicella, the percentage of anti-VZV seropositive children increased to 75% and average anti-VZV antibody concentrations increased to 250 mIU/mL at year ten after vaccination, suggesting infection or exposure. An eight-fold increase in anti-VZV antibody concentrations between two consecutive visits, which is also observed after confirmed varicella, was detected in 37% of these participants during the follow-up period. About one-third of children not vaccinated against varicella and not diagnosed with varicella developed an anti-VZV immune response, suggesting subclinical varicella occurrence. Longitudinal studies combining serology and disease incidence are necessary to reliably estimate total varicella burden of infection.

## Introduction

Infections with the varicella-zoster virus (VZV) result in varicella (chickenpox) and virus reactivation later in life results in herpes zoster (HZ; shingles).^1,2^ Varicella is a highly contagious disease, easily contracted by children and commonly regarded as a mild childhood illness.^1–4^ It is characterized by acute appearance of maculopapular vesicular skin rash, which usually recedes within a week from onset.^3–5^ Symptoms of varicella infection preceding the rash (such as fever, headache, and nausea) are usually mild in healthy children, but often accompanied by serious symptoms in adults.^3–5^ However, serious complications such as secondary bacterial infection and pneumonia can occur in all age groups as a result of varicella infection, leading to hospitalizations and, in rare cases, even to death.^2–4^

There are two main varicella vaccine formulations containing the live attenuated VZV Oka strain: the monovalent formulation (V) or the tetravalent formulation (MMRV), which combine the VZV Oka strain with the measles, mumps, and rubella (MMR) vaccine. These vaccines have proved efficacious in preventing varicella.^6,7^

Introduction of mandatory varicella vaccination in broader geographical regions has been hampered by the lack of reliable disease burden estimations.^8,9^ Varicella diagnosis relies primarily on clinical detection, based on the acute appearance of an itchy blistering rash with any number of skin vesicles as the main symptom of the disease.^2^ Previous studies also included confirmation of varicella infection via epidemiological links with valid index cases and detection of varicella virus DNA in vesicles.^10–12^ No other methods (such as serological testing of varicella antibodies) are currently recommended for varicella diagnosis or routine screening.^2^

Serological testing for varicella-specific (anti-VZV) antibodies is a complementary approach to epidemiological studies enabling the reliable estimation of total varicella infection incidence. In countries of temperate climate regions with endemic varicella, VZV seropositivity in adults is above 90%.^13^ However, these studies offer only a snapshot of the seroprevalence status^13^ and, to our knowledge, there are no prospective serological studies assessing the natural history of VZV infection in young children and its relationship with clinical disease over extended periods of time. All European studies published to date were retrospective and estimated varicella incidence through different modeling approaches.^8,9,14–16^ To date, the proportion of seropositive individuals having unknowingly experienced varicella infection remains unknown, and the contribution of such cases to the total varicella burden of infection and transmission potential is therefore difficult to measure reliably.^9,14^ In addition, the question of how anti-VZV antibody concentrations evolve over time in subclinical primary varicella cases has yet to be answered.

Previous publications from a Phase IIIb randomized trial reported that 73% of children who were not vaccinated against varicella and for whom a varicella event was not detected or reported were seropositive for anti-VZV antibodies at the end of the ten-year follow-up period.^11^ These children, included in the Active Control group to which varicella vaccine recipients (MMR+V and MMRV groups) were compared, received two doses of MMR vaccine in their second year of life.^10–12^

In the present post-hoc analysis of this Phase IIIb trial focusing on the aforementioned Active Control group, the immunological response profiles to natural varicella exposure and disease were evaluated in children from 1 year of age onwards. The evolution of varicella seropositivity and anti-VZV antibody concentrations were assessed in this population over a ten-year period. To our knowledge, this is the first prospective serological study extending over a ten-year period and aiming to characterize seropositivity prevalence and persistence of naturally induced anti-VZV antibodies in children not vaccinated against varicella and without overt clinical varicella in European countries with endemic varicella.

**Figure 1.**
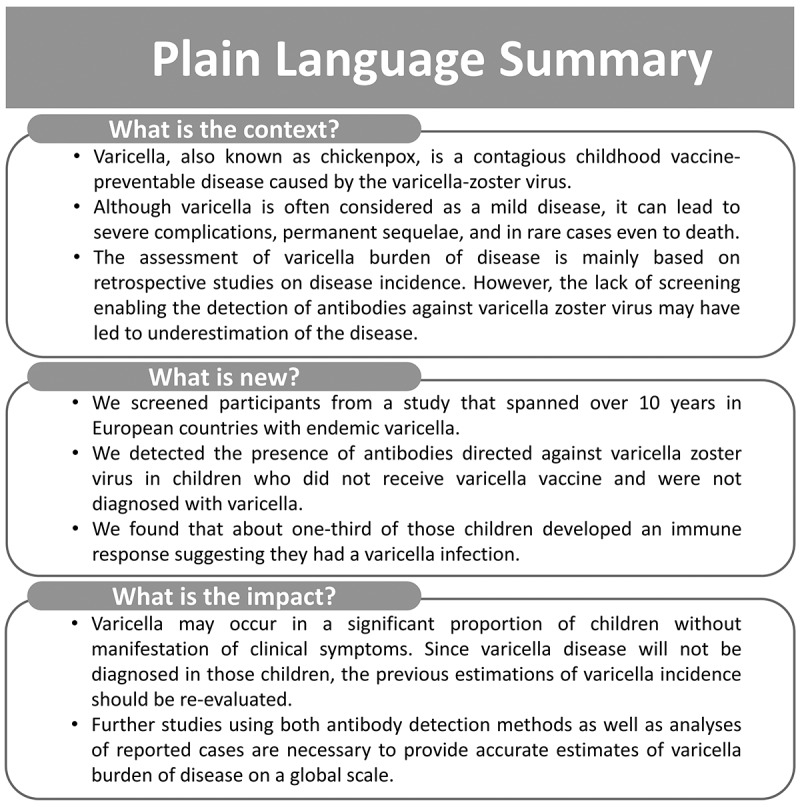
Plain language summary

A summary contextualizing the outcomes of this publication is displayed in Figure 1 for the convenience of healthcare professionals.

## Methods

### Study design and participants

**Figure 2.**
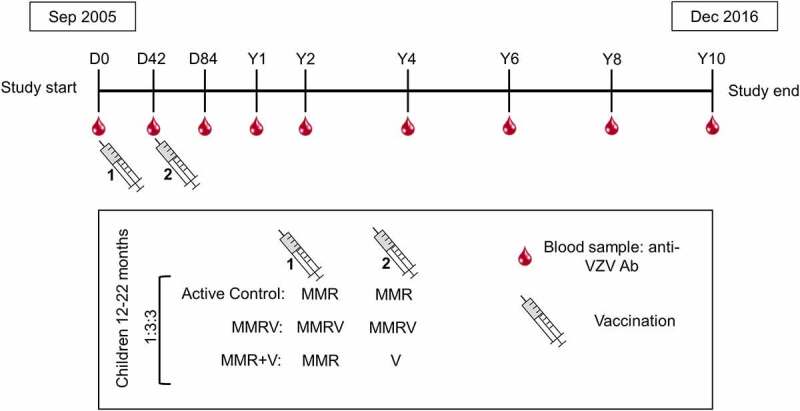
Study design

The study design was previously described in detail^11^ and is schematically presented in Figure 2. Briefly, this study was a Phase IIIb, controlled, observer-blind, multicenter, randomized study conducted in ten European countries with endemic varicella (NCT00226499). The study was conducted in agreement with the Declaration of Helsinki and followed Good Clinical Practice guidelines. It was monitored by an Independent Data Monitoring Committee (IDMC) for varicella case adjudication.^12^

D0, day of the first vaccine dose; D42, day of the second vaccine dose (Day 42, administered six weeks after the first vaccine dose); D84, follow-up timepoint at six weeks after the second vaccine dose – Day 84; Y, year; Y1-Y10, regular blood sampling and follow-up timepoints after the second vaccine dose (after one, two, four, six, eight, and ten years); Active Control, participants receiving two doses of trivalent measles-mumps-rubella vaccine (MMR); MMRV, participants receiving two doses of the tetravalent MMR-varicella vaccine; MMR+V, participants receiving one dose of MMR and one dose of monovalent varicella vaccine; 1:3:3, randomization format of participants in the treatment groups (Active control: MMRV: MMR+V); VZV, varicella-zoster virus; Ab, antibodies.

The study enrolled healthy children in their second year of life (between 12 and 22 months of age) who were followed until ten years post-vaccination. Eligibility criteria were previously described in detail.^10–12^ Parents and guardians of the study participants provided written informed consent prior to enrollment.

### Randomization and masking

Randomization was described previously and is outlined in Figure 2. Eligible participants were randomized 1:3:3 to receive two doses of MMR (Active Control group), one dose of MMR followed by one dose of V (MMR+V group), and two doses of the MMRV vaccine (MMRV group) 42 days apart (Figure 2).

The present post-hoc analysis focused on the Active Control group which remained observer-blind until completion of the study.

### Objectives

Primary and secondary objectives of the clinical trial were previously published for the follow-up periods of three, six, and ten years.^10–12^ Results of the post-hoc analysis presented here are descriptive and include the assessment of (i) the frequency of varicella occurrence, based on varicella case reports and classification, in the Active Control group, and (ii) anti-VZV antibody levels and persistence in the Active Control group.

### Procedures

#### Study vaccine and administration

All participants from the Active Control group received the same MMR vaccine lot (*Priorix*, GSK; see publication of Prymula and colleagues for details^12^) by subcutaneous injection in the deltoid region of the left arm.

#### Laboratory assessments of varicella infection and immune response

Blood samples were collected before vaccination, 6 weeks after each vaccine dose (Day 42, after dose one, and Day 84, after dose two), one and two years after the second vaccine dose and every 2 years thereafter until study completion at 10 years post-vaccination (timepoints denominated year [Y] 1, Y2, Y4, Y6, Y8, and Y10). Presence of anti-VZV IgG antibodies in the serum was assessed using the commercial enzyme-linked immunosorbent assay (ELISA) (Enzygnost, DiaSorin [formerly Siemens])^12^ and is expressed in milli-international units per mL (mIU/mL). Varicella-specific antibody concentrations measured by the anti-VZV ELISA were shown to be a reliable proxy for estimating protection against varicella in young children.^17^

In participants with suspected varicella skin eruptions, DNA was extracted from dermal vesicle samples. Extracted DNA was tested by polymerase chain reaction (PCR) test coupled with Restriction Fragment Length Polymorphism analysis of the PCR products to identify and characterize VZV serotypes. This analysis enabled the distinction between wild type and vaccine strain infections.

#### Assessment of suspected varicella cases

**Figure 3.**
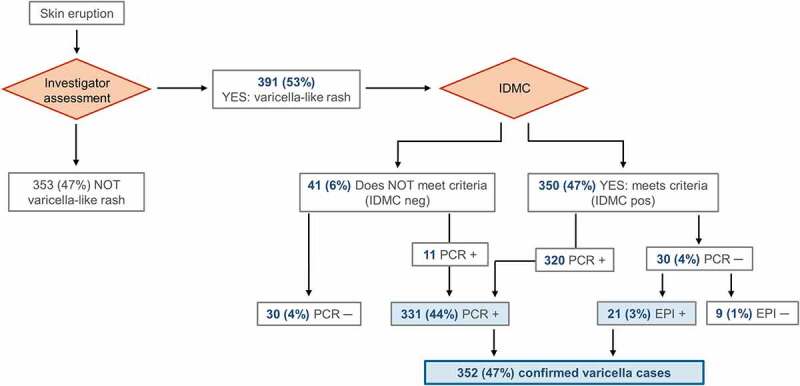
Occurrence of varicella cases in the Active Control group (per-protocol cohort for efficacy)

IDMC, Independent Data Monitoring Committee; PCR, laboratory PCR test for varicella-zoster virus DNA; EPI, established epidemiological link; +, positive; ─, negative; Cases estimated not to meet clinical case criteria by the IDMC (IDMC neg): “IDMC neg PCR ─”, suspected varicella case or no case, “IDMC neg PCR +”, PCR-confirmed, IDMC overruled case; Cases found to meet the clinical case criteria by the IDMC (IDMC pos): “IDMC pos PCR +”, confirmed varicella case, “IDMC pos PCR ─ EPI +”, epidemiologically confirmed varicella case, “IDMC pos PCR ─ EPI ─”, probable varicella case.

Reporting and evaluation of possible varicella cases was conducted as previously described^10–12^ and is presented in Figure 3. All cases of suspected varicella or HZ-like rash were promptly reported to the investigators, who arranged for clinical and laboratory assessment of vesicles. The appearance of rashes confirmed to be associated with varicella or HZ by the investigators were further reported to the IDMC for blinded evaluation if the case met the clinical definition. Rashes could also be atypical in appearance with few or no vesicles.^18^ The IDMC classified cases according to the modified scale of Vazquez and colleagues.^19^ A varicella case was considered as confirmed if it met the clinical case definition with a positive varicella PCR result, or if it met the clinical definition, was IDMC confirmed, and epidemiologically linked to a valid index case.^10–12^

### Statistical analyses

Varicella cases were followed up starting from the recorded rash onset date and were assessed in the per-protocol cohort for efficacy which included children with completed vaccinations and fulfilled protocol requirements (see Figure 3).^10–12^ Varicella antibody persistence was evaluated in the same cohort, in participants for whom valid serology results from at least two consecutive study visits were collected. The anti-VZV ELISA cutoff value was set at 25 mIU/mL, to enable distinction between seronegative children (<25 mIU/mL), seropositive children (≥25 mIU/mL) and children with seroconversion (anti-VZV antibody levels above the cutoff in previously seronegative individuals). Seroresponse was defined as an increase in anti-VZV antibody levels from <25 mIU/mL before to ≥50 mIU/mL after a defined blood sampling timepoint. The proportion of seropositive participants was calculated as the proportion of participants seronegative prior to vaccination displaying antibody concentrations above the cutoff value after vaccination. For geometric mean concentration (GMC) calculations, values below the cutoff were arbitrarily set to half the cutoff value. GMCs were calculated as the anti-log of the mean logarithmic concentration measured by ELISA.

Participants who were lost to follow-up or were withdrawn from the study were considered for the analyses up to the point of the last contact.

All computations were conducted in SAS software (version 9 · 3, with Proc-StaXact module version 8 · 1), including the calculations of 95% confidence intervals (CIs) by the method of Clopper.^20^

## Results

### Demographic characteristics

**Figure 4.**
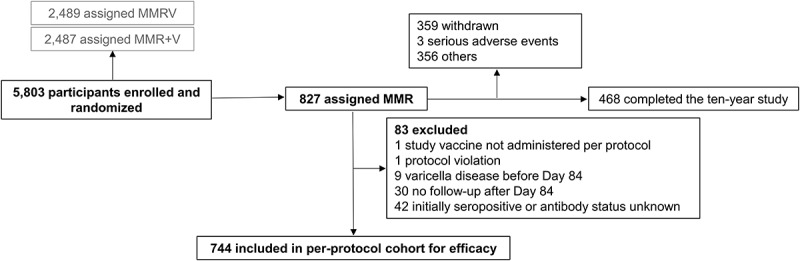
Flow of the participants included in the Active Control group

Enrollment occurred between September 2005 and May 2006. All 5,803 enrolled children were vaccinated, and 827 were included in the Active Control group (Figure 4). The per-protocol efficacy cohort included 744 children (Figure 4). The last study visit occurred in December 2016 and the median follow-up time for the entire study was ten years.

MMR, participants receiving two doses of trivalent measles-mumps-rubella vaccine (Active Control group); MMRV, participants receiving two doses of the tetravalent MMR-varicella vaccine; MMR+V, participants receiving one dose of MMR and one dose of monovalent varicella vaccine; Day 84, follow-up timepoint at six weeks after the second vaccine dose.

The demographic characteristics of the study participants were reported previously.^11,12,31^ The participant population was overall homogeneous and comparable between countries in terms of mean age, gender distribution, and ethnicity (Supplementary Table S1). Most participants were recruited in the Czech Republic, Russia, and Poland (Table 1). The mean age of enrolled children was 14 months and almost all (99%) were of European origin (Table 1). Approximately 90% of the participants had contact with other children without a known history of varicella vaccination or confirmed varicella at least once weekly.

**Supplementary Table S1:** Demographic characteristics (total vaccinated cohort) and 10-year varicella person-year rate (per-protocol efficacy cohort) of study participants per country in the Active Control group

Characteristic		Czech Republic	Greece	Italy	Lithuania	Norway	Poland	Romania	Russia	Slovakia	Sweden
**Age in months**, mean ± SD		15.3 ± 3.1	15.1 ± 2.8	13.9 ± 2.1	14.4 ± 2.2	14.1 ± 1.0	12.9 ± 1.2	15.3 ± 2.9	12.6 ± 1.2	15.4 ± 2.4	15.5 ± 2.0
**Gender**, % female		48.1	48.8	57.5	46.2	55.2	53.3	42.6	48.3	46.4	37.2
**Ethnicity**, n (%)European Arabic or North African Other		184 (99.5)	41 (95.3)	38 (95.0)	93 (100.0)	27 (93.1)	135 (100.0)	46 (97.9)	143 (100.0)	68 (98.6)	43 (100.0)
		0 (0.0)	2 (4.7)	1 (2.5)	0 (0.0)	0 (0.0)	0 (0.0)	0 (0.0)	0 (0.0)	0 (0.0)	0 (0.0)
		1 (0.5)	0 (0.0)	1 (2.5)	0 (0.0)	2 (6.9)	0 (0.0)	1 (2.1)	0 (0.0)	1 (1.4)	0 (0.0)
**Care type**, n (%)At least one sibling at homeAttending a day care centerAttending a childminderAt least once a week contact		53 (28.6)	12 (27.9)	7 (17.5)	10 (10.8)	10 (34.5)	35 (25.9)	6 (12.8)	32 (22.4)	31 (44.9)	13 (30.2)
		3 (1.6)	1 (2.3)	9 (22.5)	26 (28.0)	13 (44.8)	7 (5.2)	0 (0.0)	125 (87.4)	1 (1.4)	24 (55.8)
		13 (7.0)	9 (20.9)	7 (17.5)	1 (1.1)	8 (27.6)	4 (3.0)	0 (0.0)	12 (8.4)	4 (5.8)	4 (9.3)
		176 (95.1)	40 (93.0)	35 (87.5)	78 (83.9)	19 (65.5)	127 (94.1)	47 (100.0)	131 (91.6)	67 (97.1)	32 (74.4)
**10-year person-year rate of varicella**, n/T (95% CI)*		0.20 (0.17─0.23)	0.00 (undefined)	0.08 (0.04─0.15)	0.05 (0.03─0.07)	0.16 (0.10─0.26)	0.06 (0.04─0.08)	0.00 (undefined)	0.07 (0.05─0.10)	0.15 (0.11─0.19)	0.21 (0.15─0.30)

SD, standard deviation; n (%), number (percentage) of participants in a given category; n/T (95% CI), person-year rate (95% confidence interval). *data for Czech Republic, Lithuania, Poland, Romania and Slovakia have been published^31^

**Table 1.** Demographic characteristics of participants in the Active Control group (per-protocol cohort for efficacy; N = 744)

Characteristic		Participant features
Age in months, mean ± SD		14 ± 3
Gender, % female		48
Ethnicity, n (%)	European	737 (99)
	Arabic or North African	2 (<1)
	Other	5 (1)
Country, n (%)	Czech Republic	171 (23)
	Greece	32 (4)
	Italy	35 (5)
	Lithuania	86 (12)
	Norway	25 (3)
	Poland	116 (16)
	Romania	42 (6)
	Russian Federation	130 (17)
	Slovakia	68 (9)
	Sweden	39 (5)
Care type, n (%)	At least one sibling at home	192 (26)
	Attending a day care center	187 (25)
	Attending a childminder	57 (8)
	At least once a week contact	680 (91)

N, number of participants included in the cohort; n (%), number of participants in the defined category and their percentage relative to the corresponding cohort size; SD, standard deviation.

### Occurrence of varicella in the Active Control group (MMR recipients)

Of all reported rashes in the Active Control group, 391 (53%) were characterized as likely varicella rashes and, thereafter, assessed by the IDMC and laboratory tested. Following PCR testing and assessment of a link with a clinical index case, 39 cases (5%) were excluded as non-varicella and non-HZ cases, leaving 352 (47%) of the per-protocol cohort for efficacy participants diagnosed with a confirmed varicella case (Figure 3). The person-year rate of confirmed varicella case occurrence over the 10-year follow-up period was comparable between countries (Supplementary Table S1).

### Presence of anti-VZV antibodies in the Active Control group

**Figure 5.**
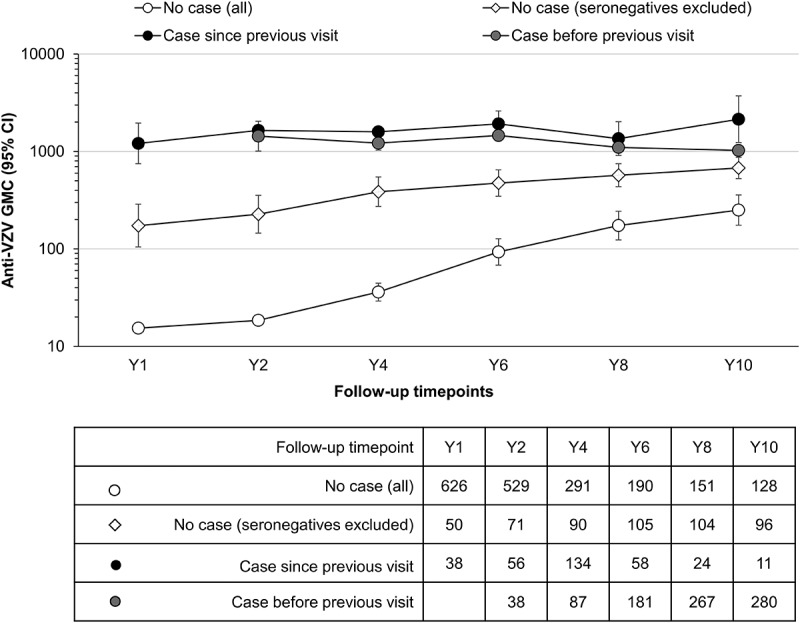
Evolution of anti-VZV antibody GMCs (lines) and the number of participants (table) in the Active Control group (per-protocol cohort for efficacy)

Anti-VZV antibody concentrations in participants from the Active Control group with and without a diagnosed varicella case were analyzed over time (Figure 5 and Table 2).

**Table 2.** Anti-VZV antibody levels in the Active Control group participants without a varicella case, or with a case detected since previous visit or before previous visit until each timepoint (per-protocol cohort for efficacy)

			Anti-VZV ≥25 mIU/mL	
Varicella case	Timepoint	N	n	% (95% CI)
No case	Y1	626	50	8 (6–10)
	Y2	529	71	13 (11–17)
	Y4	291	90	31 (26–37)
	Y6	190	105	55 (48–63)
	Y8	151	104	69 (61–76)
	Y10	128	96	75 (67–82)
Case since previous visit	Y1	38	35	92 (79–98)
	Y2	56	55	98 (90–100)
	Y4	134	133	99 (96–100)
	Y6	58	58	100 (94–100)
	Y8	24	24	100 (86–100)
	Y10	11	11	100 (71–100)
Case before previous visit	Y2	38	37	97 (86–100)
	Y4	87	86	99 (94–100)
	Y6	181	181	100 (98–100)
	Y8	267	267	100 (99–100)
	Y10	280	279	100 (98–100)

Case before previous visit, varicella case confirmed before blood sampling preceding the reference point; Case since previous visit, varicella case confirmed between previous and reference blood sampling; No case, no varicella case detected until the reference point (data for all of these participants and for a subset excluding the seronegative participants is shown); VZV, varicella-zoster virus; GMC, geometric mean concentration in milli-international units per mL (mIU/mL); 95% CI, 95% confidence interval; Follow-up timepoints, blood samples collected at indicated study visits after the second vaccine dose; Y1–Y10, post-vaccination blood samples obtained at year one, two, four, six, eight, and ten of follow-up.

One year after the second MMR dose, on average 8% of Active Control group participants without a confirmed varicella case were seropositive for anti-VZV antibodies. The percentage of seropositive participants without a diagnosed or reported varicella increased during the study. At Y10, 75% of children from the Active Control group without a confirmed varicella case were seropositive (Table 2). In participants for whom no varicella case was reported, the anti-VZV GMCs increased over the follow-up period, reaching an average GMC of 250 mIU/mL for the entire group (seronegative and seropositive participants) and 680 mIU/mL for the seropositive subgroup at Y10 (Figure 5, compare “No case [all]” and “No case [seronegatives excluded]”). When considering participants who experienced a varicella case at any timepoint, the GMC reached 1,026 mIU/mL at Y10 (see Figure 5, category “case before previous visit” at Y10). When only considering participants with a varicella case in between blood samplings, the GMC reached values as high as 2,145 mIU/mL (see Figure 5, category “case since previous visit” at Y10).

Anti-VZV antibody concentration increases for Active Control group participants with and without a confirmed varicella case were further categorized in two-fold incremental steps (two-fold, four-fold, and so on until 128-fold). When looking at increases in anti-VZV antibody concentrations between two consecutive visits, at least an eight-fold increase was detected in 97% of participants with a confirmed varicella case. A similar increase was detected in 116 out of 318 (37%) children without a confirmed varicella infection (per-protocol cohort for efficacy).

**Figure 6.**
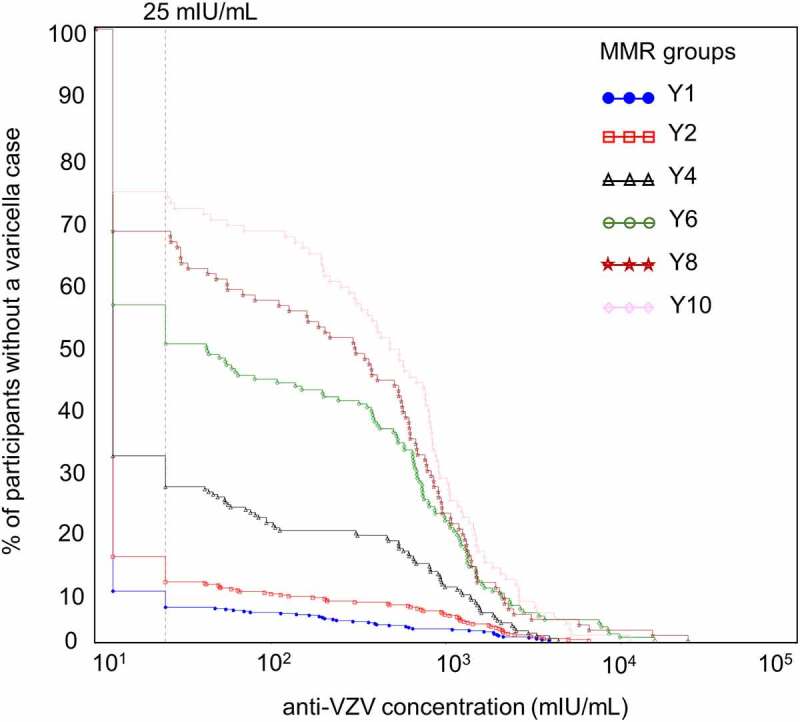
Reverse cumulative distribution curves of anti-VZV antibody concentrations in Active Control group participants without a varicella case (per-protocol cohort for efficacy)

**Figure 7.**
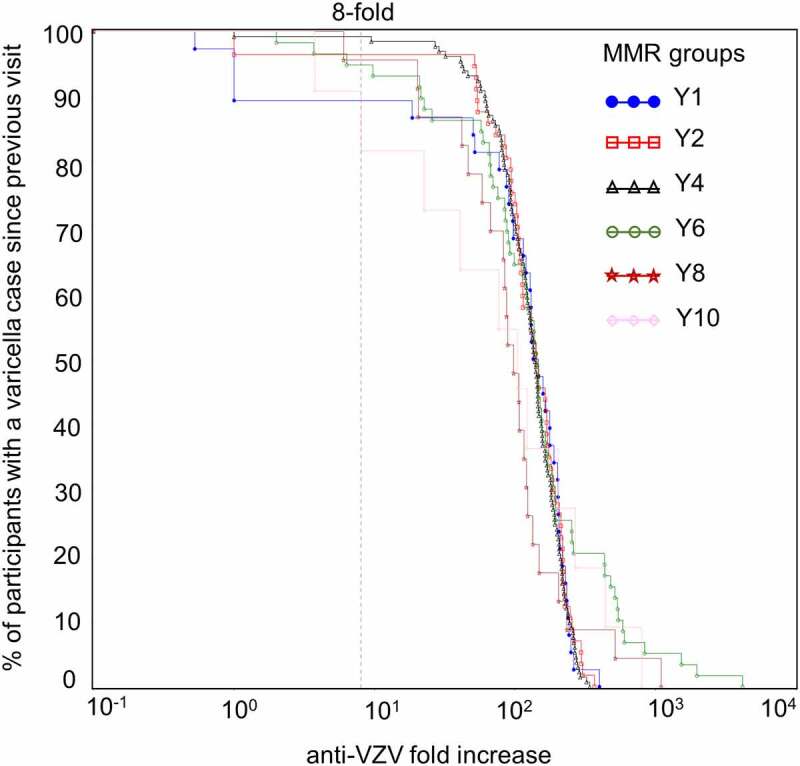
Reverse cumulative distribution curves of anti-VZV antibody concentration fold increases in Active Control group participants with a confirmed varicella case since the previous visit (per-protocol cohort for efficacy)

In children without a confirmed varicella case, there was an increase in anti-VZV seropositivity, with anti-VZV antibody concentrations spanning wide ranges (Figure 6). This increase in percentage of seropositive participants was most pronounced between Y2 and Y6 with a peak at Y4. For participants with a confirmed varicella case, comparable increases in anti-VZV antibody concentrations were observed between consecutive visits at all timepoints, regardless of when varicella cases occurred (Figure 7). The percentage of participants with a confirmed case and an increase in anti-VZV concentrations superior to 200-fold was higher at Y6, Y8 and Y10 compared to all other timepoints.

Participants without a varicella case during the ten-year follow-up period; VZV, varicella-zoster virus; mIU/mL, milli-international units per mL; ELISA, enzyme-linked immunosorbent assay; ELISA cutoff value of 25 mIU/mL of anti-VZV antibody level is indicated by the dotted line in the left panel; MMR, participants receiving the measles-mumps-rubella vaccine; Y1–Y10, post-vaccination blood samples obtained at year one, two, four, six, eight, and ten of follow-up.

Participants with a confirmed varicella case since the previous visit during the ten-year follow-up period; VZV, varicella-zoster virus; 8-fold increase in anti-VZV antibody concentration indicated by the dotted line in the right panel; MMR, participants receiving the measles-mumps-rubella vaccine; Y1–Y10, post-accination blood samples obtained at year one, two, four, six, eight, and ten of follow-up

## Discussion

This publication, reporting a post-hoc analysis of data from a Phase IIIb clinical trial with a ten-year follow-up period, describes the unexpected anti-VZV serological evolution over a 10-year period in children not vaccinated against varicella and without overt clinical varicella in countries with endemic varicella.

Previous publications from this clinical trial reported that seropositivity for anti-VZV antibodies in MMRV and MMR+V groups was characterized by a persistent and progressive increase of anti-VZV antibody levels from Y1 to Y10.^10–12^ As previously described, the GMCs at Y10 timepoint were comparable in the Active Control group without censoring of the post-infection data and the MMRV group.^11^

Overall, the participant population was nearly exclusively Caucasian (European ethnicity), and varicella person-year rate did not differ markedly between countries suggesting homogeneous exposure to circulating wild-type virus, thus making the grouping of countries for the analysis in this paper acceptable and relevant. In the present post-hoc analysis, the anti-VZV antibody levels increased in the Active Control group over several years. The percentage of anti-VZV seropositive children without a confirmed varicella case increased from 8% at Y1 to 75% at Y10. The increases in seropositivity rates and anti-VZV concentrations over time are unexpected for the Active Control group, as participants with a history of confirmed varicella were censored. Moreover, among the participants without a confirmed varicella case, throughout the ten-year follow-up, about one-third (37%) of the children experienced an eight-fold increase in anti-VZV antibody concentration between two consecutive visits, such an increase being similar to what usually happens to children having confirmed varicella. This could have been a consequence of misclassification of “no cases” (lack of clinical data or lack of household reporting) or of the presence of subclinical varicella cases. In addition, the numbers of participants without a varicella case progressively decreased during the follow-up and anti-VZV seropositivity at Y10 was observed in 96 of the 744 efficacy cohort participants. Nevertheless, these results strongly suggest that varicella infection, detected serologically by anti-VZV antibody ELISA, may occur without overt clinical skin symptoms which served as the primary method of suspicion in this and related studies.^10–12^ These undetected infection events are either truly asymptomatic, or with minimal, atypical or partial symptoms (e.g. fever only), thus not leading to a suspicion and diagnosis of varicella. While both subclinical primary VZV infections and subclinical reactivation cases have been described,^21–25^ to our knowledge, this is the first study reporting the long-term serological detection of primary VZV infection.

Overall varicella infection incidence is likely underestimated in most epidemiological surveys, as serologic screening is not systematically done. Consequently, varicella vaccine efficacy, herd protection, and benefit/risk profile may be higher than originally estimated. Furthermore, it is unknown if minimally symptomatic varicella cases may contribute to further viral transmission. It was already reported that unknown varicella or HZ disease history may hinder precise estimations of the risk of HZ occurrence.^26^ Ideally, systematic screening for presence of anti-VZV antibodies in any given population would provide valuable insight into true VZV prevalence. However, such testing should only be conducted if it is necessary to measure exposure to circulating virus and if it is deemed feasible, given the logistical and financial burden it poses to study design and its participants.

While the present post-hoc analysis was based on clinical trial data, to our knowledge, most studies on varicella prevalence are retrospective and rely on modeling to estimate the evolution of varicella seropositivity in different age groups.^8,9^ A recent review summarizing seroprevalence data from 16 European countries found that between 73% and 97% of children below the age of ten were seropositive for anti-VZV antibodies before the introduction of national varicella vaccination programs.^8^ These data are consistent with an earlier European study.^27^ A further comprehensive review of varicella seroprevalence in Europe revealed high variability in burden of disease in different countries, associated to social mixing patterns and healthcare seeking behaviors.^9^ In this review, authors estimate that, in the absence of universal varicella vaccination, the highest incidence of disease is expected in children below the age of five, with more than five million infections per year at the European level.

Other studies compared seroprevalence data with varicella case report frequencies across different age groups.^14–16^ The findings of one study from Italy indicated that varicella seroprevalence might be up to eight-fold higher than expected based solely on case reporting.^14^ The analysis was based on the discrepancy between the serological data and the reported cases. Since 2010, there are still no European standards for varicella reporting and surveillance.^28^ Therefore, future prospective and longitudinal follow-up studies would be informative to obtain accurate data on the total varicella burden of disease in Europe.

The profile of the anti-VZV antibody concentration kinetics in seropositive Active Control group participants having no confirmed varicella cases was similar to the previously published profile in MMR+V recipients.^11^ This could suggest that these participants are protected to the same level as children who received one dose of varicella vaccine. Similarly, the anti-VZV antibody concentrations were comparable between Active Control group participants with a varicella case since previous visit at Y10, obtained in this study, and the MMRV recipients at Day 84 timepoint published previously.^11^ These results are strongly supportive of a two-dose immunization schedule against varicella.

The strengths of the study include the plurality of the involved countries and centers, a long follow-up period of the same participants, and stringent controls for assessment of vaccine safety and varicella case definition.

The limitations of this study were the ethnically homogeneous population of participants and the two-year periods between serological assessments during the long-term follow-up. Despite regular contacts with the children’s families, it is possible that the time of rash onset was not always precisely determined. Another limitation was the availability of antibody concentrations only as immunological read-out, as it has been demonstrated that cellular immunity is implicated in long-term protection against varicella.^2,29,30^

To our knowledge, this is the first prospective long-term serological cohort allowing to characterize seropositivity prevalence and persistence of naturally induced anti-VZV antibodies in children not vaccinated against varicella in European countries with endemic varicella, over a ten-year period. About one-third of children not vaccinated against varicella developed an anti-VZV immune response although no varicella was detected or reported. While syndromic and seroprevalence surveys are important tools to assess the large-scale impact of varicella, used independently, such analyses are likely to reveal only part of the picture, with seroprevalence data failing to provide robust information about burden of disease and syndromic surveys potentially underestimating incidence.

## Supplementary Material

Supplemental Material

## Access to full trial protocol

For the study protocol, please see www.gsk-clinicalstudyregister.com/study100388.

## Data sharing statement

Anonymized individual participant data and study documents can be requested for further research from www.clinicalstudydatarequest.com.

## Trademark statement

Priorix is a trademark owned by or licensed to the GSK group of companies.
