# Supplementary material for: Clinically- versus serologically-identified varicella: A hidden infection burden. A ten-year follow-up from a randomized study in varicella-endemic countries
Source: Hum Vaccin Immunother. 2021 Jun 28;17(10):3747–56. doi: 10.1080/21645515.2021.1932217 (PMC8437481; doi:10.1080/21645515.2021.1932217)
Supplement: Supplemental Material [file KHVI_A_1932217_SM5191.docx]

# Supplemental material

**Supplementary Table S1: Demographic characteristics (total vaccinated cohort) and 10-year varicella person-year rate (per-protocol efficacy cohort) of study participants per country in the Active Control group**

| **Characteristic** | | **Czech Republic** | **Greece** | **Italy** | **Lithuania** | **Norway** | **Poland** | **Romania** | **Russia** | **Slovakia** | **Sweden** | |
| --- | --- | --- | --- | --- | --- | --- | --- | --- | --- | --- | --- | --- |
| **Age in months,** mean ± SD | | 15.3 ± 3.1 | 15.1 ± 2.8 | 13.9 ± 2.1 | 14.4 ± 2.2 | 14.1 ± 1.0 | 12.9 ± 1.2 | 15.3 ± 2.9 | 12.6 ± 1.2 | 15.4 ± 2.4 | 15.5 ± 2.0 | |
| **Gender,** % female | | 48.1 | 48.8 | 57.5 | 46.2 | 55.2 | 53.3 | 42.6 | 48.3 | 46.4 | 37.2 | |
| **Ethnicity,** n (%) | European | 184 (99.5) | 41 (95.3) | 38 (95.0) | 93 (100.0) | 27 (93.1) | 135 (100.0) | 46 (97.9) | 143 (100.0) | 68 (98.6) | 43 (100.0) | |
|  | Arabic or North African | 0 (0.0) | 2 (4.7) | 1 (2.5) | 0 (0.0) | 0 (0.0) | 0 (0.0) | 0 (0.0) | 0 (0.0) | 0 (0.0) | 0 (0.0) | |
|  | Other | 1 (0.5) | 0 (0.0) | 1 (2.5) | 0 (0.0) | 2 (6.9) | 0 (0.0) | 1 (2.1) | 0 (0.0) | 1 (1.4) | 0 (0.0) | |
| **Care type,** n (%) | At least one sibling at home | 53 (28.6) | 12 (27.9) | 7 (17.5) | 10 (10.8) | 10 (34.5) | 35 (25.9) | 6 (12.8) | 32 (22.4) | 31 (44.9) | 13 (30.2) | |
|  | Attending a day care center | 3 (1.6) | 1 (2.3) | 9 (22.5) | 26 (28.0) | 13 (44.8) | 7 (5.2) | 0 (0.0) | 125 (87.4) | 1 (1.4) | 24 (55.8) | |
|  | Attending a childminder | 13 (7.0) | 9 (20.9) | 7 (17.5) | 1 (1.1) | 8 (27.6) | 4 (3.0) | 0 (0.0) | 12 (8.4) | 4 (5.8) | 4 (9.3) | |
|  | At least once a week contact | 176 (95.1) | 40 (93.0) | 35 (87.5) | 78 (83.9) | 19 (65.5) | 127 (94.1) | 47 (100.0) | 131 (91.6) | 67 (97.1) | 32 (74.4) | |
| **10-year person-year rate of varicella**, n/T (95% CI)* | | 0.20 (0.17─0.23) | 0.00 (undefined) | 0.08 (0.04─0.15) | 0.05 (0.03─0.07) | 0.16 (0.10─0.26) | 0.06 (0.04─0.08) | 0.00 (undefined) | 0.07 (0.05─0.10) | 0.15 (0.11─0.19) | 0.21 (0.15─0.30) | |
| SD, standard deviation; n (%), number (percentage) of participants in a given category; n/T (95% CI), person-year rate (95% confidence interval). * data for Czech Republic, Lithuania, Poland, Romania and Slovakia have been published[^31^](#_ENREF_31) | | | | | | | | | | | |  |
